# The role of the immune system in postpartum psychosis

**DOI:** 10.1016/j.bbih.2021.100359

**Published:** 2021-09-29

**Authors:** Katie Hazelgrove

**Affiliations:** Department of Psychological Medicine, Institute of Psychiatry, Psychology and Neuroscience, King's College London, London, UK

**Keywords:** Postpartum psychosis, Immune system, Inflammation, Biomarkers, Perinatal period

## Abstract

Postpartum psychosis is the most severe psychiatric disorder associated with childbirth. The risk is particularly high for women with a history of bipolar disorder or schizoaffective disorder, or those who have suffered a previous episode of postpartum psychosis. However, the aetiology of the illness remains unclear. Pregnancy and the early postpartum are times of significant immunological change. Furthermore, alterations to the immune system have been implicated in the onset and course of various psychopathologies, both related and unrelated to childbirth. Emerging evidence, from studies on immune related disorders, immune cells and inflammatory markers, suggests that the immune system might also be involved in the pathophysiology of postpartum psychosis. Furthermore, recent research has also begun to explore the potential mechanisms underlying immune dysfunction in postpartum psychosis (e.g., disturbances in the Treg–CCN3 protein–(re)myelination axis). Nevertheless, more research is required to understand whether immune dysfunction is a cause or consequence of postpartum psychosis and to clarify the exact mechanisms involved. The aim of this short review is to present the current findings on immune system dysregulation in postpartum psychosis, discuss possible mechanisms underlying the association, highlight potential challenges and confounders and provide suggestions for future research.

## 1 Background

Postpartum, or puerperal, psychosis (PP) is the most severe psychiatric disorder associated with childbirth (Meltzer-Brody et al., 2018; Jones et al., 2014). Symptoms can begin within days of the delivery and develop rapidly (Heron et al., 2007, 2008), they can include both mania and depression, commonly in the form of fluctuating mood (Jones, 2012; Oates, 2009), hallucinations, which can be present in all sensory modalities, and delusions, which are commonly of reference, persecution or grandiosity (Spinelli, 2009; Paschetta et al., 2014). Many women with PP will also show signs of confusion and perplexity (Jones et al., 2014; Rai et al., 2015).

PP can result in considerable distress for the woman, and may have long-term consequences for their wellbeing and that of their baby and their family, as well as having implications for wider society (Jones et al., 2014). In rare cases the illness can lead to suicide and/or infanticide (Spinelli, 2009; Rai et al., 2015; Knight et al., 2018). PP is consequently considered a psychiatric emergency, typically requiring hospital admission (Jones et al., 2014; Sharma and Sommerdyk, 2014; Di Florio et al., 2013a).

PP is relatively rare, occurring in 1–2 per 1000 deliveries in the general population (Jones, 2012). However, the risk is greatly increased for women with a diagnosis of bipolar disorder, schizoaffective disorder or a personal or family history of PP, with up to 50% experiencing an episode after giving birth (Dean et al., 1989; Doyle et al., 2012; Jones and Craddock, 2001; Wesseloo et al., 2016; Di Florio et al., 2013b).

Despite the severity of PP, knowing that it has a clearly defined onset following childbirth and that women with certain pre-existing diagnoses are at increased risk, the exact causes of the illness remain unknown. Emerging evidence suggests the immune system might play a role. The aim of this short review is to discuss the current findings on immune dysregulation in PP.

## 2 The immune system and the perinatal period

Pregnancy and childbirth are times of dramatic immunological change. Indeed, it has been suggested that pregnancy represents a unique period of immunomodulation, characterised by increased recognition, communication, trafficking and repair (Mor et al., 2011; Racicot et al., 2014). This unique and highly dynamic immune response is essential for establishing and maintaining pregnancy, with the maternal immune system promoting tolerance towards the semi-allogenic foetus, whilst also providing protection from pathogens. To achieve this, it has been proposed that pregnancy involves three distinct immune stages, characterised by both pro- and anti-inflammatory states (Mor et al., 2011; Racicot et al., 2014). The first stage is necessary for successful implantation and placentation. Immune cells, including natural killer (NK) cells, dendritic cells and macrophages, infiltrate the decidua and surround the invading trophoblasts. However, rather than rejecting the blastocyst, these immune cells instead play a supportive role, repairing and restructuring uterine tissue (Mor et al., 2011, 2017; Maguire et al., 2020; Leff-Gelman et al., 2016; Sherer et al., 2018). The first and early second trimester of pregnancy are, therefore, characterised by a T helper (Th) 1-type proinflammatory response, necessary to repair damaged tissue and remove cell debris (Mor et al., 2011, 2017; Maguire et al., 2020; Dutta and Sengupta, 2017). Following successful implantation and placentation, there is a period of rapid foetal growth and development, which lasts from pregnancy weeks 13–27 (Mor et al., 2011, 2017). In order to maintain pregnancy, and prevent miscarriage or preterm birth during this phase, there is a switch from the Th1 proinflammatory environment to a predominantly Th2-type anti-inflammatory state (Mor et al., 2011, 2017). In addition to promoting immune tolerance towards the foetus, anti-inflammatory responses in this stage also promote uterine growth and foetal development (Mor et al., 2017; Sherer et al., 2018; Dutta and Sengupta, 2017). Thus, the second immunological stage provides a supportive immune environment in which symbiosis is established between maternal and foetal physiology (Mor et al., 2011, 2017; Maguire et al., 2020; Dutta and Sengupta, 2017). Once foetal development is complete, the final immunological stage of pregnancy occurs in preparation for the delivery. Following the onset of labour, immune cells migrate into the myometrium resulting in a shift back to a proinflammatory state. This proinflammatory milieu promotes uterine contractions and the subsequent expulsion of the baby and placenta (Mor et al., 2011, 2017; Maguire et al., 2020). Finally, pre-pregnancy immune function is not thought to return for up to several months post-delivery (Groer et al., 2015).

Whilst essential for the pregnancy and for foetal development, it is suggested that these changes in immune physiology during the perinatal period can contribute to maternal perinatal psychopathology, particularly in women with an existing vulnerability (Yim et al., 2015). Research has indeed shown immune system dysregulation in women with perinatal depression. For example, our group recently showed elevated immune markers (Interleukin (IL)-6, IL-10, Tumor Necrosis Factor (TNF)-α and Vascular Endothelial Growth Factor) in pregnant women with a diagnosis of major depressive disorder, compared with healthy pregnant controls (Osborne et al., 2018). Furthermore, there is some evidence to suggest that a heightened proinflammatory response, including increased IL-6, IL-1β and TNF-α, is associated with postnatal depressive symptoms (Maes et al., 2000; Boufidou et al., 2009; Corwin et al., 2008; Liu et al., 2016).

There is also research showing that immune system dysregulation plays a role in the aetiology of psychopathology unrelated to gestation. Indeed, altered immune function has been implicated in the pathophysiology of bipolar disorder and psychosis unrelated to gestation, with research consistently showing elevations in various immune markers (e.g., IL-1β, sIL-2, IL-6, TNF-α and C-reactive protein (CRP)) in individuals with psychosis (Zajkowska and Mondelli, 2014; Miller and Goldsmith, 2019; Miller et al., 2014) and those with bipolar disorder (Pereira et al., 2021). Furthermore, there is emerging evidence that elevated immune markers could be predictive of subsequent illness (Miller and Goldsmith, 2019; Khandaker et al., 2014; Osimo et al., 2021).

Given that pregnancy and childbirth are times of immunological change (Mor et al., 2011, 2017), and the fact that immune dysregulation has been linked to other psychopathology (e.g., Osborne et al., 2018; Boufidou et al., 2009; Liu et al., 2016; Zajkowska and Mondelli, 2014; Pitharouli et al., 2021), it is indeed plausible that the immune system might play a role in the pathophysiology of PP.

## 3 The immune system and PP

### 3.1 Immune-related disorders and PP

Emerging evidence for the role of the immune system in PP has come from studies examining immune-related disorders in women with PP. In an observational study of primiparous women, Bergink and colleagues examined the association between autoimmune thyroid dysfunction and first-onset PP (Bergink et al., 2011a). The authors found that autoimmune thyroid disease (AITD) was more prevalent in women with first-onset PP than postpartum women from the general population, at both 4 weeks' (prior to initiation of antipsychotic and mood stabiliser treatment) and 9 months' postpartum. Furthermore, the authors reported a faster and greater progression to clinical thyroid dysfunction in women with PP *and* AITD, when compared with the postpartum control group (Bergink et al., 2011a).

These findings are consistent with an earlier case report, which documented the simultaneous onset of postpartum thyroiditis and PP in a 29-year-old woman with no past history of psychiatric or general medical disorders (Bokhari et al., 1998). Simiarly, a previous study of thyrotoxicosis and hypothyroidism in postpartum women found evidence of PP-like symptoms in the women with postpartum thyroid dysfunction. The authors reported that the psychotic symptoms improved when the women became euthyroid again (Amino et al., 1982).

More recently a nationwide register-based cohort study examined the bidirectional association between first-onset AITDs and first-onset psychiatric disorders, including PP (Bergink et al., 2018). The study found that, after adjustment for covariates, women with first-onset postpartum AITDs were at increased risk of experiencing a first-onset psychiatric disorder compared with those who did not have postpartum AITDs and vice versa (i.e., first-onset postpartum psychiatric disorders were associated with an increased risk of AITDs). Furthermore, high comorbidity was found between first-onset AITDs and psychiatric disorders in the postpartum period (Bergink et al., 2018). Given the autoimmune aetiology of AITD, these findings provide evidence to support the hypothesis that immune dysfunction plays a role in the pathophysiology of PP.

Simiarly, in another population-based cohort study, Bergink and colleagues examined whether pre-eclampsia, considered a disease of immunological maternal-foetal incompatibility, is a risk factor for postpartum psychiatric episodes (Bergink et al., 2015). The study found that pre-eclampsia increased the risk of a psychiatric episode, including PP, in primiparous women without a psychiatric history. This is in line with earlier findings by Bergink and colleagues, who reported a higher incidence of pre-eclampsia during pregnancy in women who subsequently developed an episode of PP compared with postpartum controls (Bergink et al., 2011b). However, a more recent population-based cohort study, examining the association between pregnancy and obstetric-related complications and risk for different types of first-onset postpartum psychiatric disorders, found no association between pre-eclampsia and PP (Meltzer-Brody et al., 2017). Given these mixed findings, additional research is needed to confirm whether pre-eclampsia increases the risk of PP.

### 3.2 Immune cells and PP

Evidence of immune system dysregulation in PP also comes from studies examining the status of various immune cells. For example, Bergink and colleagues carried out a comprehensive analysis of immune cells in women with first-onset PP compared with healthy postpartum and non-postpartum women (Bergink et al., 2013). They found that, compared with non-postpartum controls, postpartum controls showed elevated T cells, present across various T cell subsets (including Th1, Th17 and natural Regulatory T cells (Tregs)). On the other hand, women with PP did not show this typical postpartum increase in T cells, but instead had significantly lower T cells (both total T and Th1 cells) compared with healthy postpartum women (Bergink et al., 2013). More recently, examining subpopulations of T cells, Kumar and colleagues found that women with PP had lower naïve CD4 and CD8 cells, but higher activated CD8 and memory Tregs, compared with healthy postpartum women (Kumar et al., 2017). Taken together, these findings suggest that increases in T cells are part of normal immune function during the postpartum period and the inability of women with PP to show these typical postpartum increases appears to emphasise the role of various T cell subsets in the pathophysiology of the disorder.

Monocyte levels have also been found to be altered in women with PP. Indeed, Bergink and colleagues found increases in total monocyte levels, as well as upregulation of monocyte gene expression, in the women with first-onset PP, compared with both healthy postpartum and non-postpartum women (Bergink et al., 2013). Kumar and colleagues on the other hand examined subtypes of monocytes and found that women with PP showed a decline in non-classical monocytes compared with healthy postpartum women (Kumar et al., 2017). Further evidence for the relevance of monocyte alterations in PP comes from research showing downregulation of microRNA (miR)-146a and miR-212 expression in the monocytes of women with PP. Interestingly, miR-146a expression was decreased in the monocytes of women with first-onset PP, while miR-212 was decreased in the monocytes of women with PP *and* a history of bipolar disorder, possibly signifying an inherent difference between episodes of PP limited to the postpartum period and those occurring in the context of a diagnosis of bipolar disorder (Weigelt et al., 2013). Weigelt and colleagues also found that downregulation of miR-146a expression in monocytes was associated with reduced natural Tregs in women with PP (Weigelt et al., 2013), which further fits with the findings of a reduction in T cells in women with PP (e.g., Bergink et al., 2013; Kumar et al., 2017).

As well as altered T cell and monocyte levels, Kumar and colleagues found that, compared with healthy postpartum women, women with PP showed alterations in NK cells (Kumar et al., 2017). The authors reported a marked decrease in cytotoxic NK cells, which they propose is suggestive of a possible defect in normal postpartum immune restoration. They also found a significant increase in regulatory NK cells, which are thought to be involved in NK cell cytotoxicity suppression during the postpartum period (Kumar et al., 2017). Finally, dendritic cells have also been implicated in PP. Kumar and colleagues found a reduction in myeloid dendritic cells in women with PP when compared with healthy postpartum women (Kumar et al., 2017). Taken together the findings suggest PP is characterised by immune dysregulation, involving both pro- and anti-inflammatory processes.

### 3.3 Inflammatory markers and PP

More recently, research has also examined levels of inflammatory markers (e.g., cytokines) in PP. Indeed, Sathyanarayanan and colleagues investigated cytokine and chemokine changes in women with first-onset PP compared with healthy postpartum and non-postpartum women (Sathyanarayanan et al., 2019). Interestingly, IL-6 was found to be elevated in both the women with PP and the healthy postpartum women, compared with those in the non-postpartum group. The authors propose that this could indicate that increased IL-6 is part of a normal postpartum biological response. In line with this finding, our own research group found no difference in levels of IL-6 between women with PP, those at risk (because of a diagnosis of bipolar disorder, schizoaffective disorder or a history of PP) who were well in the postpartum period and healthy postpartum controls (Aas et al., 2020), supporting the idea that IL-6 does not represent a pathophysiological marker for PP.

On the other hand, Sathyanarayanan and colleagues found elevated levels of IL-8 in women with PP, compared with healthy postpartum women (Sathyanarayanan et al., 2019). Furthermore, the authors found that healthy postpartum women had similar levels of IL-8 to those in the non-postpartum control group, suggesting that IL-8 might be a characteristic of immune system dysfunction in PP (Sathyanarayanan et al., 2019). Additionally, our research group found that women with PP had increased high sensitivity (hs) CRP (a more precise measure, which can detect lower levels of CRP) when compared with healthy postpartum women. Interestingly, we found that women at risk of PP who were well in the postpartum period had hsCRP levels that were intermediate between those of women with PP and healthy postpartum women, suggesting that hsCRP might represent a trait marker for severe postpartum mental illness, which is then exacerbated in those women who become unwell after giving birth (Aas et al., 2020). Of note, a recent systematic review and meta-analysis of 21 studies found that elevated CRP was associated with increased risk of suicidality, and particularly suicidal ideation, in individuals with psychiatric disorders, including those with affective and non-affective psychoses (Miola et al., 2021). This is particularly relevant given that PP is associated with an increased risk of suicide, which remains one of the leading causes of maternal death (Jones et al., 2014).

In addition to IL-8 and hsCRP, previous research has reported increased expression of Monocyte Chemoattractant Protein (MCP)-1 levels in women with first-onset PP, compared with both healthy postpartum and non-postpartum women (Bergink et al., 2013), suggesting that MCP-1 might also be a marker of immune dysregulation in the illness. However, this finding was not replicated in subsequent studies (Sathyanarayanan et al., 2019; Aas et al., 2020). Given this inconsistency in findings, the association between MCP-1 and PP requires further investigation.

Table 1 provides an overview of the three immune molecules found to be associated with PP (i.e., IL-8, CRP and MCP-1), including an outline of their function and associations with other psychiatric disorders, both related and unrelated to gestation.

**Table 1:** Overview of the key molecules associated with PP.

Molecule	Abbreviation	Description	Function	Associations with other psychiatric disorders
Interleukin 8	IL-8/CXCL8	Proinflammatory chemokine produced by a wide range of cell types, including macrophages, epithelial and endothelial cells, in response to inflammation.	Plays a key role in activation and migration of neutrophils, as well as basophils and T cells, to sites of infection.	Evidence to suggest IL-8 is elevated in patients with psychosis unrelated to gestation (e.g., Di Nicola et al., 2013; Frydecka et al., 2018; Kamińska et al., 2001; Reale et al., 2011). Some evidence to suggested increased IL-8 and IL-8/IL-10 ratio are associated with postnatal depression (e.g.,Achtyes et al., 2020; Corwin et al., 2015).
Monocyte Chemoattractant Protein-1	MCP-1/CCL2	Proinflammatory chemokine produced by a variety of cell types, including macrophages, epithelial and endothelial cells, in response to inflammation.	Induces the recruitment and activation of monocytes, but also the migration of T cells and NK cells, to sites of infection or tissue injury.	Evidence to suggest MCP-1 is elevated in patients with psychosis unrelated to gestation (e.g., Frydecka et al., 2018; Hong et al., 2017; Klaus et al., 2021; Orhan et al., 2018). Lack of research examining MCP-1 in postnatal depression.
C-Reactive Protein	CRP	Acute-phase protein produced by the liver in response to inflammation, infection and trauma.	Binds to the surface of pathogens and dead or damaged cells. This activates the complement system, promoting phagocytic activity, which helps to eliminate the pathogens and dead/damaged cells.	Evidence to suggest CRP is elevated in patients with psychosis unrelated to gestation (e.g., De Berardis et al., 2013; Devanarayanan et al., 2016; Fawzi et al., 2011). Mixed findings in postnatal depression, with some studies finding elevated CRP in women with postnatal depression (e.g., Liu et al., 2016), whilst others report no association between CRP and postnatal depression (e.g., Albacar et al., 2010; Miller et al., 2019; Simpson et al., 2016).

## 4 Immune system dysregulation as a predictor of PP

The above research provides support for the hypothesis that PP is associated with immune dysfunction. However, the majority of studies conducted to date have used cross-sectional designs, which cannot determine the direction of the association, i.e., whether immune system alterations are a cause or a consequence of PP. Longitudinal studies, which can disentangle directionality, are therefore needed to address the question of cause and effect in the association between immune system dysfunction and PP. Establishing whether immune dysfunction is present prior to illness onset could help predict which women are most likely to become unwell in the postpartum period.

Our research group recently attempted to investigate this in a prospective longitudinal study of women at risk of PP (because of a diagnosis of bipolar disorder, schizoaffective disorder and/or a previous history of PP). Women were recruited in pregnancy and followed-up to 4 weeks' postpartum, in order to ascertain who did and did not have a psychiatric relapse in the postpartum period. Immune markers were assessed in the 3rd trimester of pregnancy (Hazelgrove et al., 2021) (also discussed in Hazelgrove (2021)). Contrary to what we expected, women at risk of PP who went on to have a psychiatric relapse in the first 4 weeks' postpartum did not differ from the women at risk who remained well in the postpartum period in their levels of antenatal inflammatory markers (including, IL-1β, IL-6, IL-8, TNFα or hsCRP). In fact, we found that both at-risk groups had similar mean cytokine levels as healthy pregnant women. This finding is perhaps surprising given the accumulating evidence of immune alterations in women with PP. Given that previous research has been conducted in women who had already developed an episode of PP, one possible explanation is that while immune system dysregulation might be a characteristic of PP, it is not necessarily present prior to illness onset. It is, however, important to note that we used a broader definition of PP (i.e., psychiatric relapse), which captured relapse of all affective symptoms in the immediate postpartum (including depressive symptoms). It is, therefore, possible that immune dysfunction, while not a marker for more broadly defined postpartum episodes in women at risk of PP, could be predictive of full-blown PP. However, given that PP is a polymorphic clinical entity, characterised by various symptoms, including depression and mania, it is possible that we would not have found differences even if full-blown PP had been assessed. Whilst it was not possible to explore this in our research, it warrants further investigation.

Interestingly, we did find that MCP-1 levels in the third trimester of pregnancy were lower in women at risk of PP who relapsed following delivery, compared with women at risk who remained well. Although only reaching a trend for significance, this might suggest that MCP-1 has a ‘protective’ effect on the risk of postpartum relapse in women at risk of PP. This would appear to contradict previous findings of increased MCP-1 in women with PP (Bergink et al., 2013). This could be because we measured MCP-1 levels during pregnancy before illness onset, whereas Bergink and colleagues assessed women in the postpartum period during an acute illness state (Bergink et al., 2013). However, as discussed, other studies have not found altered MCP-1 in women with PP (Sathyanarayanan et al., 2019; Aas et al., 2020). On the other hand, our findings are in line with the literature on treatment-resistant depression, which found depressed patients who did not respond to pharmacological treatment had lower levels of MCP-1 at baseline than responsive patients (Carvalho et al., 2013). However, given that MCP-1 was the only inflammatory marker to differ between the women at risk who relapsed and those who remained well, and that the difference only reached a trend for significance, it is important to interpret the finding with caution. Additional research in larger samples is recommended to gain a better understanding of the role of MCP-1 in the development of PP.

## 5 Mechanisms underlying the association between immune system dysregulation and PP

It has been suggested that immune system dysregulation could play a role in the onset of psychotic disorders via both direct pathways (e.g., autoimmunity or infection) and more complex, immune-mediated, pathways (for review see Bergink et al. (2014)). While the exact mechanisms underlying the association between immune dysregulation and PP remain unclear, one hypothesis recently proposed by members of our group is that immune system involvement in PP may occur through immune system-mediated myelination processes, namely disturbances in the Treg–CCN3 protein–(re)myelination axis (Dazzan et al., 2018). Specifically, the authors propose that women at risk of PP are more likely than healthy women to show disturbances in Treg activity and/or abundance, which in turn could be associated with altered CCN3 protein expression and subsequently with reduced (re)myelination in cortical and limbic brain regions (Dazzan et al., 2018).

In support of this hypothesis, our research group has found preliminary evidence of reduced myelin content in the temporal lobes and sublobar regions of women at risk of PP compared with healthy postpartum controls (Giordano et al., 2017). Further evidence for the link between abnormalities in myelination processes and risk for PP comes from a case report showing lesions in the splenium of the corpus callosum, the largest white matter structure in the brain (Udaya et al., 2015). There is also evidence for the involvement of CCN3 in PP. Indeed, in a pharmacological mouse model of PP, Humby and colleagues showed increased CCN3 expression in the brain tissue of new mouse mothers that were administered a steroid sulfatase inhibitor (Humby et al., 2016) (used to mimic maternal steroid sulfatase deficiency, which has been proposed as a potential risk factor for PP (Davies, 2012)). Interestingly, when examining the expression of immune-related genes whose activity has been shown to be regulated by CCN3 (e.g., Le Dréau et al., 2010), Humby and colleagues found increased MCP-1 gene expression in the brains of the steroid sulfatase-inhibited mice (Humby et al., 2016), which supports the findings of increased MCP-1 expression in serum of women with first-onset PP (Bergink et al., 2013).

The exact mechanisms by which peripheral disturbances in Treg activity and/or abundance lead to altered CCN3 expression, and by which CCN3 expression might influence (re)myelination processes, remain unclear. Dazzan and colleagues suggest that it could be possible for Tregs to enter the brain through permeabilization of the blood-brain barrier, possibly facilitated by increased expression of MCP-1 (Dazzan et al., 2018), which enhances blood-brain barrier permeability (Yadav et al., 2010; Yao and Tsirka, 2014). Indeed, immune dysfunction has been found to be associated with blood-brain barrier compromise, leading to an influx of unwanted immune cells and inflammatory molecules into the brain (Lee and Giuliani, 2019; Huang et al., 2021). Nevertheless, additional research is needed to confirm whether disturbances in the Treg-CCN3-(re)myelination axis play a role in the aetiology of PP and, if so, the precise mechanisms underlying these processes.

## 6 Potential challenges and confounders

It is of course important to consider the findings outlined above considering the potential challenges and confounders associated with studying the immune system in postpartum women, and particularly those affected by psychiatric illness.

Firstly, given that there are changes to the immune system over the postpartum period, with ‘normal’ non-pregnant immune state thought to return around 3–4 months' post-delivery (Groer et al., 2015), the time (i.e., number of days/weeks/months) postpartum at which immune function is assessed could influence findings. Several of the studies discussed above have reported between group differences in number of weeks after delivery at the time of assessment. For example, in the study by Sathyanarayanan and colleagues, women with PP were recruited up to 6 weeks' postpartum, whilst healthy postpartum women were recruited up to 12 weeks' postpartum (Sathyanarayanan et al., 2019). There was also a difference in time post-delivery in our recent research by Aas and colleagues, with women with PP assessed significantly later than healthy women or those at risk of PP (Aas et al., 2020). However, after controlling for number of weeks post-delivery Aas and colleagues found that women with PP continued to have increased hsCRP compared with healthy postpartum women, suggesting that time post-delivery could not fully explain the immune findings (i.e., elevated hsCRP in women with PP) (Aas et al., 2020). Nevertheless, it is important for future studies examining immune function in PP to consider the possible influence of time post-delivery.

It is also important to consider breastfeeding status, which is thought to influence immune function. For example, a study by Groer and colleagues found a heightened immune activation in postpartum women, which was further enhanced in those who exclusively breastfed (Groer et al., 2005), whilst a study by Ahn and Corwin found lower levels of IL-6 at 6 months' postpartum in women who were primarily breastfeeding, compared with those who primarily bottle fed (Ahn and Corwin, 2015). Furthermore, exclusive breastfeeding might be more likely in healthy postpartum women than those with PP. Indeed, in their study of immune system function in PP, Bergink and colleagues found that postpartum controls were significantly more likely to breastfeed than women with PP (Bergink et al., 2013). Simiarly, the studies by Kumar and colleagues and, more recently, Sathyanarayanan and colleagues reported that whilst *all* women in the healthy postpartum group exclusively breastfed, those in the PP group gave a mixture of breast- and formula-feeding (Kumar et al., 2017; Sathyanarayanan et al., 2019). However, despite these differences in breastfeeding status, Bergink and colleagues found no association between breastfeeding and T cell or monocyte activation in their study (Bergink et al., 2013). Nevertheless, most studies of immune function in PP do not appear to have examined the relationship between breastfeeding and immune function. Thus, it is not possible to rule out the influence of breastfeeding on immune status in PP.

Another perinatal-specific factor shown to be associated with immune function is mode of delivery. Indeed, Bergink and colleagues found women with PP who had a cesarean section had increased MCP-1 levels, whilst healthy postpartum women who had a cesarean section had elevated percentages of Th1 and Th2 helper cells, compared with those who had a vaginal delivery (Bergink et al., 2013). The authors also reported group differences in mode of delivery, with healthy postpartum women being more likely to have had a cesarean section than those with PP (Bergink et al., 2013). Interestingly, the studies by Kumar and colleagues and Sathyanarayanan and colleagues found that a higher percentage of women with PP had a cesarean section (25% of women with PP, compared with 5% of healthy postpartum women) (Kumar et al., 2017; Sathyanarayanan et al., 2019). Future studies in PP should, therefore, control of the effects of mode of delivery on immune function.

Finally, research has shown psychotropic medication to have an impact on immune function, including evidence of both pro- and anti-inflammatory effects of various psychotropic treatments (Baumeister et al., 2016). Despite this, not all studies investigating the immune system in PP have controlled for psychotropic medication use, potentially limiting the interpretation of the findings. Of the two studies that did consider medication use, neither found it to be associated with altered immune measures (Bergink et al., 2013; Aas et al., 2020). However, one of the studies reported that most women with PP had only been taking medication for a few days at the time of the assessment (Bergink et al., 2013), making it difficult to draw firm conclusions about the impact of longer-term medication use on immune function in PP.

## 7 Conclusion and future directions

There is growing evidence to suggest that immune dysfunction is a core feature of PP. However, the research to date provides little evidence to suggest that these alterations in immune function are present prior to the onset of PP and, therefore, predictive of the illness, particularly in women at risk. Nevertheless, the majority of studies have examined immune function in women *with* PP, thus more research in large prospective samples is needed to understand whether immune dysfunction represents a biological marker for PP. On the other hand, there is evidence to suggest that episodes of PP limited to the postpartum period might be biologically distinct from those occurring in the context of bipolar disorder (e.g., Weigelt et al., 2013). This could provide information relevant to the diagnosis and treatment of PP and, therefore, merits further investigation. Finally, hypotheses around the potential mechanisms linking immune dysfunction to PP have also been proposed (e.g., disturbances in the Treg–CCN3 protein–(re)myelination axis (Dazzan et al., 2018)). However, more research is required to clarify the precise mechanistic processes involved. Ultimately, understanding the biological underpinnings of PP has important implications for both potential treatments (e.g., the ability to develop targeted treatments) and early identification of those most at risk (e.g., closer monitoring of primiparous women who experience pre-eclampsia or AITD).

## Funding

This work was supported by the UK Medical Research Foundation [grant number C0439]. The research was also in part supported by the UK Medical Research Council [grant number MR/s003444/1, e-BRAIN Study] and the UK National Institute for Health Research (NIHR) 10.13039/100014461Biomedical Research Centre at South London and Maudsley NHS Foundation Trust and King's College London. The funding sources played no role in writing the article or in the decision to submit the article for publication.

## Declaration of competing interest

The author declares that they have no known competing financial interests or personal relationships that could have appeared to influence the work reported in this paper.
